# Shaping Behaviors Through Institutional Support in British Higher Educational Institutions: Focusing on Employees for Sustainable Technological Change

**DOI:** 10.3389/fpsyg.2020.584857

**Published:** 2020-12-03

**Authors:** Fuqiang Zhao, Fawad Ahmed, Muhammad Khalid Iqbal, Muhammad Farhan Mughal, Yuan Jian Qin, Naveed Ahmad Faraz, Victor James Hunt

**Affiliations:** ^1^School of Management, Wuhan University of Technology, Wuhan, China; ^2^Knowledge Unit of Business, Economics, Accountancy and Commerce, University of Management and Technology Sialkot Campus, Sialkot, Pakistan; ^3^School of Management Sciences, Tianjin University of Finance and Economics, Tianjin, China; ^4^Birmingham City Business School, Birmingham City University, Birmingham, United Kingdom

**Keywords:** intention to use, education management information systems, self-efficacy, institutional support, personal innovativeness, organizational support theory

## Abstract

Technology permeates all walks of life. It has emerged as a global facilitator to improve learning and training, alleviating the temporal and spatial limitations of traditional learning systems. It is imperative to identify enablers or inhibitors of technology adoption by employees for sustainable change in education management systems. Using the theoretical lens of organizational support theory, this paper studies effect of institutional support on education management information systems (EMIS) use along with two individual traits of self-efficacy and innovative behavior of academic employees in British higher educational institutions. Data for this cross-sectional study were collected through a questionnaire completed by 591 academic employees of 23 universities from 10 cities in the United Kingdom. Partial Least Square structural equation modeling was used to analyze data with smartPLS 3.2.9 software. Results indicate that institutional support promotes self-efficacy and innovative behavior that help develop positive employee perceptions. The model explains a 52.9% variance in intention to use. *Post-hoc* mediation analysis shows that innovativeness and self-efficacy mediate between institutional support and employee technology adoption behavior. As opposed to student samples in past studies on educational technology, this study adds to the literature by focusing on academic employees.

## Introduction

Technology has emerged as a global facilitator to improve learning and training, alleviating the temporal and spatial limitations of traditional learning systems. Two tendencies in educational institution research are prevalent; one, digital competence in curriculum design and assessment methods (Buitrago Flórez et al., 2017), and the other is encouraging teachers to integrate technology to facilitate learners (Scherer et al., 2019). Educational management information systems (EMIS) have attained unprecedented practical significance (Martins et al., 2019). The significant amounts of data created in this digital age need to be structured, adequately analyzed, and presented with accuracy for the public to benefit from it. This notion can also be translated to the field of education because education management has become an increasingly complex process from the institutional perspective of managing resources and business. The use of EMIS has increased because it provides quality information and key functionality in higher education institutions (Haris et al., 2017). EMIS is not merely used for storing information; rather generates new information and knowledge as well. It makes institutions more competitive in their performance and success toward students’ learning. This calls for the need to adopt such technology by academic employees.

Academic employees are at the helm of every successful technological change process in educational institutions since implementation requires positive employee attitudes as a unit of change (Ahmed et al., 2019). This process is also dependent on granting employees access to perceived organizational support, which enables and empowers them in technology use. The organizational support theory (Eisenberger et al., 1986) posits that employees reciprocate support received through positive behavior and actions. The role of perceived organizational support has been proven to moderate the attitudes of employees (Khan et al., 2017). Especially if they need support to enhance computer self-efficacy (Liu et al., 2020) when using EMIS. Support for employees enhances their traits like innovativeness and level of self-efficacy, which manifests employee perceptions (Ratchford and Barnhart, 2012). The same applies to EMIS for employees’ propensity to adopt the technology. Mercader and Gairín (2020) found that institutional factors (e.g., lack of training, knowledge of online teaching techniques, and planning) were among the top barriers in university teachers’ use of digital technologies. Several studies have found that individual drivers, such as personal innovativeness and a higher level of self-efficacy, positively affected the adoption of technologies such as m-learning (Briz-Ponce et al., 2017; Oskay, 2017). With respect to readiness for organizational change, Holt et al. (2016) suggest that readiness for change depends, in part, on the “change-specific-efficacy” of employees and the level of management support extended to the employees.

Most research efforts have focused on digital technologies from a learner perspective, and rather little is known about the role of perceived organizational support and employees at such universities that have the capacity to implement online education management systems. A plethora of studies is available on numerous kinds of organizations for technology upgrades, customers’ behavior, employee attitudes, and organizational change as a whole for varying technologies (Rahayu and Day, 2015; Alalwan et al., 2016; Atkin et al., 2017; Ukpabi and Karjaluoto, 2017; Zainab et al., 2017; Alzahrani et al., 2018; Chuang, 2018; Roy et al., 2018). Interestingly, there is a barrage of studies on educational technology adoption, but most of it focuses on student samples, and little is known about the drivers of employee attitudes toward the use of technology in institutions (Nistor et al., 2014). However, the literature on employees’ technology usage behaviors at educational institutions is scarce (Scherer et al., 2019). In blended learning environments with online systems, the teacher must continue to provide active consultation (Gregori et al., 2018), and hence their continued technology use is imperative.

This paper offers fresh insights to the application of organizational support theory by focusing on academic employees in British higher education institutions. It studies how workplace social exchange construct of institutional support influences the individual constructs of innovativeness and self-efficacy toward EMIS use. A robust review of peer-reviewed literature failed to uncover any previous research that investigates these as collective predictors of the technology acceptance model (TAM). Scherer et al. (2019) stressed the need of further research to “shed more light on the processes of technology acceptance and extend the current perspective of the TAM.” Research on the implementation of educational technologies is moving slowly in developing countries because most research tends to focus on the driving factors that affect these systems (Al-Emran et al., 2020). There is a scarcity of studies on the teacher’s role as an employee in online educational technology environments (Ross et al., 2014). Recent systematic reviews on technology acceptance studies reveal a lack of regard for employees and academicians as users in the implementation of educational technology (Granić and Marangunić, 2019; Al-Emran et al., 2020). Moreover, there is a scarcity of studies on the role of perceived organizational support in EMIS adoption behavior amongst academic employees. Based on the arguments above, the objectives of this study are:

• To examine if institutional support has an influence on academic employees’ intention to use EMIS.
• To ascertain whether institutional support predicts self-efficacy and personal innovativeness of employees.
• To determine if self-efficacy and personal innovativeness affect employee perceptions and intention to use EMIS.

With these objectives in mind, this paper aims to provide empirical evidence to support the factors forming part of a process that affects employee intention to adopt EMIS using an extended (TAM). Not only does this study fill a gap in the literature, but it also responds to a call for research in a meta-analysis by Scherer et al. (2019). To that end, this study contributes in multiple ways; first, it integrates organizational support theory (Eisenberger et al., 1986) with self-efficacy and innovativeness; and extends the TAM model from an employee’s perspective, whilst considering organizational as well as individual-level antecedents, which is a first. Second, up until the time this study was conducted, employees, including both teachers as well as non-teaching staff, have not been studied collectively as users of EMIS in any British university work setting; most studies have focused on student samples or only on teachers for a specific technology. This paper also aims to respond to Al-Emran et al. (2020) more recent call for research on academics’ behavior toward educational technology use. Third, a review of literature on TAM from 1986 to 2013 by Marangunić and Granić (2015) has revealed that previous studies have not used true “older” adult samples since most respondents were relatively young and have thus called for more studies to be conducted with older participants. More than 52% of respondents of this study were aged 46 or above and thus representing true older adults. Fourth, Al-Emran et al. (2020) recently called for research that included samples from multiple institutions nationwide to ensure better generalizability of TAM. This study fills that gap by including data from 23 universities spread country-wide and offers better generalizability. No such study has been conducted on EMIS use; prior research largely constitutes data collected from a single educational institution at one point in time; whereas, this study included multiple institutions and used temporal separation in data collection to address possible common method bias.

The remainder of the paper proceeds as follows. The next section discusses the relevant concepts about EMIS and related information systems from the existing literature; explaining the research model and hypotheses; followed by methodology explaining procedures and measures used in this study to collect and analyze the data. Then the results are explained along with the model strength and quality, which follows a discussion on relevant implications. Finally, concluding remarks are made including the limitations of this study and suggestions for future research.

## Literature Review and Hypotheses

### Education Management Information Systems—EMIS

Theoretically, EMIS have been seen as information systems used for producing, managing, and disseminating educational data and information as a routine matter under existing I.T. infrastructure (Tolley and Shulruf, 2009). The initial conceptualizations have been merged with the theoretical concepts and practical characteristics on this topic, such as by Cash (2015); Bessa et al. (2016), and Martins et al. (2019). This merger of conceptualizations has allowed for general perceptions to evolve beyond customary views and for the use of EMIS to be seen under a dual perspective: (1) Its use by management at an institutional level to look for information required to make strategic decisions and by teachers to manage resources and assessment process, and (2) Its use by students for individual learning; collecting and analyzing information required to make decisions regarding learning-related activities; and for interaction with other stakeholders such as professors and class fellows (Holsapple and Lee-Post, 2006).

Martins et al. (2019) build a strong argument that the term EMIS constitutes a range of systems used in academic settings. As observed by Cash (2015), EMIS have been studied under varying conceptual terms, although mostly from student perspectives. These include virtual learning systems (Nistor et al., 2014), Learning Management Systems (LMS) (Al-Emran et al., 2020), virtual learning environment (Cao et al., 2019), Mobile Learning (m-learning) (Al-Emran et al., 2018), student engagement in educational technology (Bond et al., 2020), web-based learning (Yi and Hwang, 2003) or simply Management Information Systems (MIS) (Martins et al., 2019). EMIS used in educational institutions hold similar significance as that of Enterprise Resource Planning (ERP) software applications used in other forms of organizations. Some educational institutions in the United Kingdom use customized versions with a combination of EMIS, LMS, m-learning, and OGS features (Hook et al., 2015; Bond et al., 2020). The purpose of these systems includes but is not limited to, real-time processing of and access to information such as study materials, students records, assessment, and teaching resources; real-time grading of assignments; removal of physical barriers to resources; removal of the dependence on the physical presence of both the respective faculty and students; and removal of the distance between non-teaching and teaching staff for better administrative coordination. One of the key factors in bringing about operational efficiency is the ability of organizations to process dynamic data and information and to create service innovation to make EMIS successful for consumers, i.e., students and management (Martins et al., 2019). This requires employees with innovative abilities and high self-efficacy.

### Institutional Support, Self-Efficacy, and Innovative Behavior

According to organizational support theory (Eisenberger et al., 1986), support extended to employees invokes a perspective of social exchange in which employees feel obliged to repay the organization with better input through extra roles such as innovative behavior. The same holds true for employee intentions to use new technology at the workplace. Past research has also shown that institutional support could be an antecedent to ease of use and usefulness Davis et al. (1989). Management Support has a proven impact on technology acceptance (Lee et al., 2013). Rhoades and Eisenberger (2002) opine, “Institutional support is generally characterized by the organization’s legal, moral, and financial responsibility … and by the power, the organization’s agents exert over individual employees.” Training sessions can be held pre-as well as post-implementation and can benefit employees’ learning and continued use of technology. The availability of I.T. computing technical support should help with quick troubleshooting, leading to a fast and significant increase in employee confidence in adopting new technology. Policies of institutional support significantly assist users in their information systems usage behavior, leading to an accumulation of knowledge and experience (Lin and Hwang, 2014) and the resultant understanding and clarity on the use and value of the system, thus producing positive perceptions.

Self-efficacy is a psychological belief of an employee in his/her ability. Compeau and Higgins (1995) concluded in their study on ICT that self-efficacy can be characterized as our perception of our potential to use technology. Their study proved that support to users positively affects computer self-efficacy. Management support has proven to enhance employee commitment and result in positive attitudes in employees (Lee et al., 2017). Scherer et al. (2019) argue in their meta-analysis that self-efficacy is a strong predictor of core variables in TAM and can be either a barrier or enabler in technology adoption. They further comment, “yet, the direct or indirect mechanisms leading up to this importance are still to be examined in greater detail.” They suggest “training approaches targeted at… may also focus on enhancing teachers’ self-efficacy in using technology.”

In situations where firms set up a broader mechanism at an organization-level to allocate enough resources in order to provide support for the adoption of any given system, they face a lesser number of constraints in terms of resource allocation or basic provisions to enable such adoption (Ratnasingam, 2005). There is a contradictory viewpoint amongst researchers who have found an adverse relationship between resources allocated for the support of a change initiative of system adoption. Internal resource competition is one of the more frequently identified hurdles within the organizational units (Tsai, 2002), which, in turn, could create a negative effect on innovation. Institutional support aims at removing such hurdles that inhibit innovative behavior.

Thus we hypothesize:

**H1a:** Institutional support (IS) will positively affect self-efficacy (SE) of employees.
**H1b:** Institutional support (IS) will positively affect intention to use (IU) EMIS among Employees.

### Self-Efficacy and Technology Adoption Behavior

Self-efficacy has been shown to predict PEU and PU (Lee et al., 2013). Usher and Pajares (2008) observed that self-efficacy is one of the significant factors in determining what degree of effort a person shall put in during the performance of any behavior. Similarly, Wilson et al. (2007) observed that people with higher self-efficacy have a higher chance of success in any given assignment. Eastin and LaRose (2000) argue that this concept originates from the studies on Internet Communication Technologies (ICT) self-efficacy. Lin and Hwang (2014) investigated the relationship between an individual’s influence on innovation and technology adoption and found that self-efficacy and personal perceptions about the ability to create knowledge using I.T. has a direct influence on affective commitment (Doǧru, 2017; Jaradat et al., 2018).

Studies on the relationship between self-efficacy and perceived ease of use are contradictory in their results. Many have proven self-efficacy to be a strong predictor of perceived ease of use. At the same time, some studies have shown that self-efficacy has no significant impact on technology usage behavior (Venkatesh et al., 2003; Ozturk et al., 2016). Moreover, self-efficacy has also been studied as a moderator for various variables in their interaction with PEU (Jaradat et al., 2018). Individuals with self-efficacy have confidence in their ability to operate the new technology and are more likely to adopt new technology (Ratchford and Barnhart, 2012; Al-Haderi, 2013). On the other hand, such individuals who think that the use of technology is too complex and that they are not capable of operating it on their own are likely to reject such technology’s use.

**H2:** Self-Efficacy (SE) of employees will have a positive effect on Perceived Ease of Use (PEU) of EMIS.

### Innovation Diffusion Theory

Rogers (1983) has defined diffusion in his Innovation diffusion theory as “the process through which an innovation reaches the members of any social system via varying channels.” Rogers put forth a combination of perceptions, and (Tidd and Bessant, 2005; Besson and Rowe, 2012) provided an extended version of that proposed set of perceptions through inclusion of seven further factors associated with an innovation serving as independent variables for IT acceptance. It is improbable that each one of the employee could be as innovative as the other and it is equally impossible that every employee would possess the innovativeness trait. As Hwang (2014) showed in their study, personal innovativeness is a leading cause of creating perceptions of ease among users of IT. They further discovered that this relationship is in fact moderated prior experience of the users. So, if there are more innovative people in a team of employees, then it has higher possibility of receiving acceptance for any new given innovative technology and that too shall be very fast in terms of implementation time.

As literature defines it, innovativeness is “a process through which an individual (or other decision-making unit) passes from first knowledge of an innovation to forming an attitude toward the innovation, to a decision to adopt or reject, to implementation of the new idea, and to confirmation of this decision” (Rogers, 1983). Rogers has also divided the adopters into early adopters and late adopters, the TAM is used for assessing intention to use EMIS because it is more relevant to early adopters and early majority. There are other models established over time, e.g., UTAUT and TAM 3, which address such factors influencing adoption of technology which would be more relevant to late adopters in the long run.

### Personal Innovativeness and Technology Adoption

**FIGURE 1:**
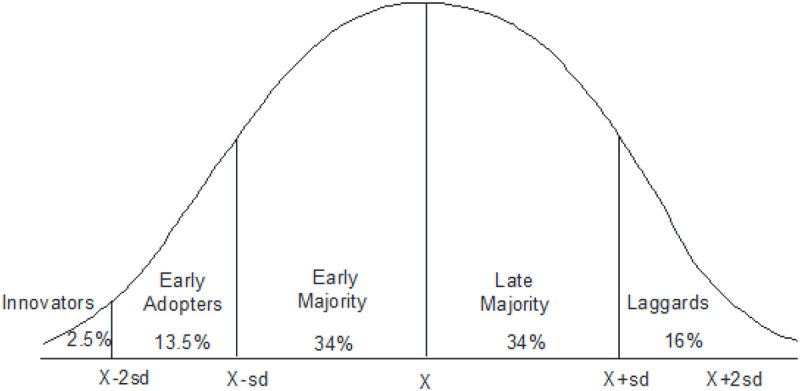
Process of innovation adoption (Rogers, 1983).

Innovation diffusion theory by Rogers (1995) explains innovativeness as the tendency of individuals to be quicker than their peers in accepting new technology (Figure 1). Crompton and Burke (2018) opine that user’s learning can be affected by a number of variables. One such variable is the user’s ability to handle a technology effectively, in other words, innovativeness. Not all employees have an innovative disposition. As a personality trait, it helps shape individual perceptions about one’s ability to cope with changes; to understand intricate technical knowledge, and to deal with the uncertainty that comes with any new technology. While reviewing the relevant literature, it has been observed that numerous personality traits have been studied for their effect on the technology adoption by individuals in varying backgrounds and contexts such as insecurity, optimism, discomfort, innovativeness, self-efficacy, trust, and perceived risk, etc. (Liljander et al., 2006; Erdoǧmuş and Çiçek, 2012; Lai et al., 2012; Ahmed et al., 2019).

Personal innovativeness of British respondents proved to be a direct predictor of intention to use mobile payments (Slade et al., 2015). The direct effect of innovativeness on perceived usefulness has been confirmed for ERP systems to be user acceptance as well (Hwang, 2014). Joo (2015) found no influence of innovativeness on perceived usefulness and called for more research suggesting the possibility of other factors involved in both personal innovativeness and usefulness. Hwang (2014) found that personal innovativeness leads to a perception of usefulness amongst users, and it is moderated by user experience. So, if a team of workers has more people with innovative behavior, a given innovation of new technology is presumed to find acceptance and implementation more quickly. The more an individual is innovative, the more likely it is for him to embrace challenges and be inclined toward technology adoption. Therefore, innovativeness develops a more positive attitude toward the adoption of technology (Kabra et al., 2017). A study observed that personal innovativeness had a significant effect on the user’s intentions toward m-learning adoption (Mahat et al., 2012). Contrary to the majority of the studies, Agarwal and Karahanna (2000) observed that perceived usefulness was not affected by personal innovativeness.

Previous works include some studies that measured technology readiness and technology acceptance by studying innovativeness as a predictor (Walczuch et al., 2007; Caison, 2008). These have demonstrated that more innovative users display positive attitudes toward the use of that technology irrespective of whether it is easy to use or not. They do not care if the technology in question is useful for their job-tasks or not, but deem it useful because of the innovativeness trait and perceive it useful as an opportunity to try out a new system. The results of a study by Mahat et al. (2012) on mobile learning in higher education showed that respondents displaying moderately high innovativeness also displayed higher technology acceptance. Similarly, it can be argued that academic employees with a higher level of innovativeness will have a higher tendency to use EMIS.

Therefore we hypothesize:

**H3a:** Personal innovativeness (INN) of employees will positively affect their Perceived Usefulness (PU).

Personal innovativeness, as a trait and predictor, is an individual’s willingness to try out relatively new information technology and can help identify early adopters (Agarwal and Prasad, 1998). Social exchange theory, which is based on organizational support theory, posits that employees tend to show extra-role behaviors such as innovativeness as a result of the care and support received from management. Khan et al. (2017) studied factors affecting the adoption of LMS and found that institutional support played a moderating role in creating positive employee behavior. Because of its potential predictive power, institutional support has been used as an enabler and enhancer of self-efficacy and innovativeness, as it provides an improved understanding of the adoption and use of information systems. It can be logically argued that in any given educational setting, a pro-active management approach that extends support to employees should result in resources abundance. This should also encourage more users to adopt technology progressively.

**H3b:** P**e**rsonal innovativeness (INN) of employees will mediate the positive relationship between institutional support (IS) and intention to use (IU) EMIS.

### Technology Acceptance Model (TAM)

TAM is one of the most powerful, robust and parsimonious model for predicting user acceptance especially in IS context (Bueno and Salmeron, 2008; Qin and Ahmed, 2017). Past works include some studies to measure technology readiness and technology acceptance (Walczuch et al., 2007; Caison, 2008). Scherer et al. (2019) observed that TAM is repeatedly indicated in literature as one of the most powerful, robust and parsimonious models in order to predict user acceptance, particularly in context of information systems. In order to predict and elaborate the user’s adoption behavior and acceptance for any give technology, there has been ample research conducted in past to ascertain the determinants of acceptance and use of information technology and systems. Generic Theory of Reasoned Action (TRA) holds as the primary stimulant for further research as IT explained a user’s attitude toward technology (Im et al., 2011; Magsamen-Conrad et al., 2015; Chauhan and Jaiswal, 2016).

**FIGURE 2:**
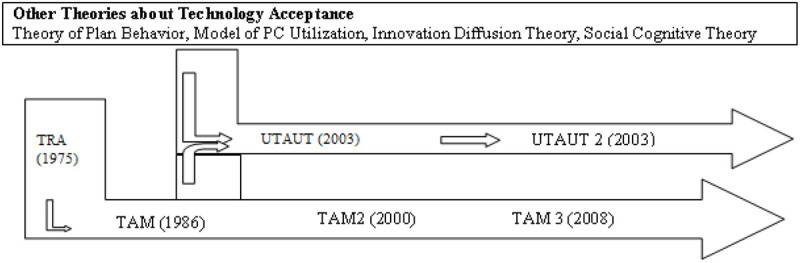
Evolution of TAM. Source: Im et al. (2011); Magsamen-Conrad et al. (2015), and Chauhan and Jaiswal (2016).

TRA has successfully argued that an individual’s behavior can be predicted by behavioral intention. Working on the same lines, researchers developed a similar model, namely Technology Acceptance Model (TAM), which has been used as an even more prevalent model. TAM has been adapted from TRA. Ngai et al. (2014) argue TAM is the culmination of the underlying concepts of TRA and Theory of Planned Behavior (TPB) (Figure 2). Fishbein and Ajzen (1975) developed the TRA which has proven as one of the most used models to study intention and is deemed as suitable “to explain virtually any human behavior” Ajzen and Fishbein (1980).

The key variables of TAM (Davis, 1985) are perceived usefulness (PU), perceived ease of use (PEU), and intention to use (IU). This study uses TAM because it has been tested empirically and supported through validations, applications, and replications (Venkatesh and Davis, 2000). Davis et al. (1989) defined PU As a potential user’s personal opinion that using a specific technology may be a deciding factor in the achievement of their goals. The TAM originally maintains that the inner beliefs of individuals regarding the usefulness and the ease of use of technology are significant predictors of its adoption and continued use. Daǧhan and Akkoyunlu (2016) studied the use of m-learning systems in public universities for continued intention to use. They found the user’s perceived value and usability as antecedents of intention to use m-learning information systems. PEU is believed to have an indirect effect through PU onto IU, Moreover, there is also evidence-based research on online LMS and virtual academic communities (Nistor et al., 2014).

**FIGURE 3:**
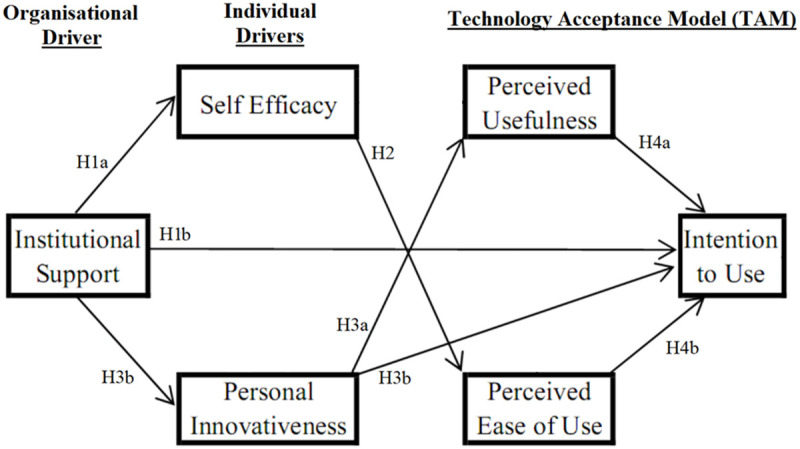
Research model.

Therefore, we hypothesize a significant path from both PU and PEU to IU Perceived usefulness has repeatedly proven to affect attitude (Figure 3). Kim and Rha (2018) explored factors influencing the users’ intention toward adopting m-learning in Korea and observed that perceived usefulness as well as the users’ attitudes toward its use played a significant role as determinants of m-learning adoption. Lee et al. (2013) observed it to be a direct determinant of continued intention to use information systems. However, there are not many studies showing employee readiness at universities in United Kingdom. This study will test the core TAM and hypothesizes as below:

**H4a:** Perceived usefulness (PU) of EMIS will be positively associated with intention to use (IU).
**H4b:** Perceived ease of use (PEU) of EMIS will be positively associated with intention to use (IU).

## Research Methods

This cross-sectional study draws on quantitative research to examine the proposed relationships. A questionnaire survey was conducted to test the hypotheses developed in the previous section.

### Sampling and Data Collection

The sample was drawn using probabilistic sampling from high-ranking universities in the United Kingdom. There are 169 degree-awarding higher education recognized in the United Kingdom (Gov.UK, 2020). Numerous universities were shortlisted through the Quacquarelli Symmonds World Rankings for the year 2020. The official website lists 1,002 top universities worldwide. These included 84 universities from the United Kingdom. Top universities were chosen for their more substantial focus on innovative practices and early technological upgrades, as compared to other smaller specialized universities. The authors reached out to personnel in managerial roles for liaison at these 84 target universities through email to request participation. 39 universities replied with indication of willingness to participate. Two of the authors visited these 39 willing universities to meet the management in person and explained the purpose of this study to seek permission for data collection and eventually, 23 agreed to participate after permission was granted by authorized personnel for data collection. The distribution of the questionnaire was left at the discretion of liaison persons at the universities because they had the first-hand knowledge about which academic employees are actually using the EMIS and would understand the questionnaire so as to avoid unnecessary waste of time.

For determining the sampling frame, which can be defined as “a list of elements from which a sample may be drawn; also called working population” (Zikmund et al., 2013). The authors ensured that sample chosen covers the population as a whole (Lindner et al., 2001). The universities in the United Kingdom are clustered into four groups by Boliver (2015) and five clusters by Research England in a report by Ulrichsen (2018). The sample for this study included universities from all geographical parts of Great Britain and also included at least one university from each of the clusters categorized by both Boliver (2015) and Research England.

The questionnaire explained the purpose and voluntary nature of the study and that there was no right or wrong answer. In order to maintain anonymity, the employees were assigned codes for questionnaires, and no personally identifiable information was obtained. Temporal separation (Podsakoff et al., 2003) was observed by collecting data in two waves. Questionnaires were distributed in October 2019 to each of the key liaison persons at target universities and were collected back in December 2019. Inclusion criteria were provided to the liaisons, which required that all respondents must be permanent employees and have worked with the organization for at least 1 year. The authors prepared two survey packets labeled Survey 1 and Survey 2. Survey 1 contained items for IS, INN, and SE and Survey 2 constituted a questionnaire for TAM variables. In the first wave, 866 academic employees were invited to participate in this study, and 696 respondents filled and returned the questionnaire to the liaison at each university from October 14 to November 1, 2019. Out of these, 672 (77.59%) were considered viable responses after screening for missing data. Two weeks later, from November 17 to December 2, 2019, liaison persons asked these 672 respondents to fill in Survey 2. Out of these, 609 responded, while 18 responses were not usable. Responses of both surveys were combined using the codes assigned to employees in the beginning to match data sets for the full research model. Eventually, 591 (68.24%) responses were deemed usable for data analysis.

The questionnaire items were randomly arranged and mixed instead of grouping statements for each variable in continuity. Moreover, Harman’s test (Podsakoff et al., 2003) was also carried out, and it proved that common method effects did not contaminate the results as no single factor emerged with high variance. Eight factors emerged with Eigen values greater than 1, accounting for 68.3% variance, and the highest variance by any one single factor was 16.2%, which is well below the 25% threshold. For non-response bias, the early (first 50%) and late (second 50%) respondents of survey 2 were compared and there were no significant differences found between the two groups for any of the paths in research model. Demographics were compared between 81 non-respondents of Survey 2 who had initially responded to survey 1 and the 591 respondents of survey 2 and there were no differences found.

The respondents constituted 347 (63.20%) males and 244 (36.80%) females; 22 (5.6%) were under 30 years of age while 264 (41.9%) were between 30 and 45 years of age and remaining 305 (52.5%) were older than 45 years. Given the nature of their jobs, respondents were highly educated, and over 56.02 percent (*n* = 331) had a Ph.D., 43.99% (*n* = 260) had a masters degree; 273 (46.19%) had over 5 years experience while 318 (53.81%) had less than 5 years experience; 148 (25.05%) were full professors, 109 (18.44%) associate professors, 103 (17.43%) assistant professors and 231 (39.08%) lecturers.

In order to ensure the adequacy of the sample size, the researchers followed the recommendations of Hair et al. (2013) that the required sample size should be based on a power analysis considering that part of the model which has the largest number of predictors. In this study, the most number of arrows (predictors) pointing at a dependent variable was 04 predictors pointing toward intention to use. The minimum sample size for 80% power, significance level of 5%, and a minimum 25% *R*^2^-value is 65 observations (Wong, 2013).

### Control Variables

Past literature shows varying results for the effect of age and gender in technology adoption behavior. Venkatesh et al. (2003) also found differences in technology usage behavior between men and women. More recently, Okumus et al. (2018) found that users showed differences in mobile diet app usage on the basis of their gender. Therefore, this paper undertakes to study the differences, if any, in usage behavior among respondents based on age, gender, experience and job role through multi-group analysis (MGA).

### Measures

The questionnaire (Appendix Table A1) consisted of 23 items. Perceived Usefulness (five items) and Perceived Ease of Use (five items) and Intention to Use (three items) were each measured through statements modified from Davis et al. (1989) and Venkatesh and Davis (2000). Innovativeness (four items) was measured through statements adopted from Agarwal and Prasad (1998), more recently used by Cao et al. (2019). Institutional Support (three items) was measured through statements modified from Mathieson et al. (2001), more recently used by Park et al. (2014) and Kazumi and Kawai (2017). Self-efficacy (three items) was measured through statements adopted from Taylor and Todd (1995) and Igbaria and Iivari (1995). All measures were self-assessment types and were anchored by a seven-point Likert scale ranging from “strongly disagree (1)” to “strongly agree (7).” A pilot study was carried out with 30 responses from three universities. The results proved the questionnaire to be a valid and reliable tool for conducting this research. Questionnaires from several previous studies were adapted for this study to ensure content validity and reliability. Questions were re-worded for this study.

### Data Analysis

The data analysis was carried out using SmartPLS 3.2.7 (Ringle et al., 2015). PLS was the preferred option because PLS can deal with issues related to skewness and multi-collinearity robustly. SmartPLS is a preferred option for prediction is the main purpose of the study. It is also a more appropriate technique for complex models (Hair et al., 2019). Therefore, the reason for choosing PLS over other software applications was its ability to estimate the relationship between multiple independent and dependent constructs of structural models and the latent, multiple observed or unobserved constructs of measurement models, simultaneously. Smart-PLS software application uses a variance-based SEM approach. This was an advantage over other covariance-based (CB-SEM) software (e.g., SPSS, AMOS) which requires separate treatments of data to address skewness and multi-colinearity problems and is not suitable for simultaneous estimation of multiple variables in a prediction-oriented research model. One of the main advantages of PLS-SEM over CB-SEM is that PLS-SEM can handle numerous independent variables at the same time even when they have multicollinearity.

## Results

The results are analyzed in two steps. First, the measurement model is assessed, followed by an assessment of the structural model (Sarstedt et al., 2017).

### Measurement Model Assessment

The measurement model was assessed by evaluating item reliability, construct reliability and validity through reflective indicators. The item reliability of each latent variable was assessed with the minimum cut-off criterion for the loading of an item (indicator) set at 0.5. The item loading for the majority of the indicators exceeded the value of 0.70 (see Table 1) and ranged between 0.505 and 0.90. However, one item of PU with an item loading of below 0.50 was removed.

**TABLE 1:** Cross loadings, construct reliability and convergent validity.

**Items**	**Cross loadings**						**CR**	**AVE**
	**INN**	**IU**	**IS**	**PEU**	**PU**	**SE**		
INN1 <- INN	0.664	0.344	–0.094	0.096	0.257	0.193	0.818	0.531
INN2 <- INN	0.695	0.269	0.038	0.111	0.273	0.236		
INN3 <- INN	0.826	0.455	0.105	0.242	0.252	0.468		
INN4 <- INN	0.720	0.403	0.080	0.090	0.214	0.311		
IU1 <- IU	0.416	0.812	0.442	0.485	0.207	0.419	0.829	0.622
IU2 <- IU	0.428	0.889	0.238	0.243	0.131	0.497		
IU3 <- IU	0.387	0.645	–0.072	0.029	0.115	0.162		
IS1 <- IS	0.069	0.200	0.731	0.216	0.180	0.429	0.803	0.571
IS2 <- IS	0.048	0.285	0.798	0.301	0.022	0.327		
IS3 <- IS	–0.021	–0.015	0.505	–0.304	–0.212	0.068		
PEU1 <- PEU	0.142	0.122	0.267	0.811	0.306	0.373	0.883	0.604
PEU2 <- PEU	0.136	0.202	0.185	0.706	0.375	0.409		
PEU3 <- PEU	0.178	0.186	0.269	0.883	0.408	0.402		
PEU4 <- PEU	0.104	0.244	0.176	0.736	0.389	0.282		
PEU5 <- PEU	0.224	0.304	0.220	0.737	0.303	0.452		
PU1 <- PU	0.399	0.291	0.118	0.380	0.737	0.362	0.840	0.569
PU2 <- PU	0.179	0.137	0.032	0.405	0.805	0.384		
PU3 <- PU	0.057	–0.089	0.056	0.416	0.750	0.092		
PU5 <- PU	0.333	0.198	0.159	0.327	0.723	0.396		
SE1 <- SE	0.443	0.423	0.296	0.306	0.279	0.776	0.838	0.634
SE2 <- SE	0.300	0.438	0.467	0.330	0.394	0.869		
SE3 <- SE	0.336	0.463	0.188	0.279	0.334	0.739		

**CR, composite reliability of construct; AVE average variance extracted.**

The internal consistency reliability of constructs was established through composite reliability (C.R.), which is a more appropriate measure for reliability as compared to Cronbach’s alpha since C.R. uses weighted items. All the constructs showed high composite reliability scores (Table 1) ranging between 0.803 and 0.883, thus suggesting sufficient reliability.

The convergent validity was assessed with the average variance extracted (AVE) values following suggestions by Fornell and Larcker (1981). The AVE values for all the constructs were greater than 0.5 and ranged between 0.531 and 0.710, thus confirming that more than 50% of the indicator’s variance is explained by the construct and hence proves convergent validity.

The discriminant validity (Table 2) was assessed through Fornell-Larcker as well as heterotrait-monotrait (HTMT) criteria developed by Henseler et al. (2014) using ratios of the correlations to assess the discriminant validity. HTMT for all variables was below the conservative threshold of 0.85, thus, confirming discriminant validity (Sarstedt et al., 2017). The square root of AVE values (in diagonal) is higher than all other values in the relevant columns below the diagonal, thus confirming discriminant validity (Fornell and Larcker, 1981). Table 3 displays estimates of loadings and significance (generally termed as confirmatory factor analysis) with *t*-statistics and significance values for each item.

**TABLE 2:** Discriminant validity.

	**INN**	**IS**	**IU**	**PEU**	**PU**	**SE**
INN	0.729*	0.170	0.822	0.261	0.458	0.593
IS	0.066	0.756*	0.435	0.493	0.318	0.567
IU	0.607	0.262	0.789*	0.513	0.332	0.789
PEU	0.200	0.288	0.325	0.777*	0.702	0.616
PU	0.334	0.125	0.193	0.576	0.777*	0.590
SE	0.442	0.418	0.548	0.486	0.576	0.796*

*Square root of AVE in italics, HTMT values above the diagonal.

**TABLE 3:** Confirmatory factor analysis.

**Items**	**Original sample (O)**	**Mean**	**SD**	***T*-statistics (O/SD)**	***P*-values**
INN1	0.662	0.652	0.069	9.602	< 0.001
INN2	0.693	0.694	0.060	11.642	< 0.001
INN3	0.827	0.828	0.031	26.618	< 0.001
INN4	0.723	0.713	0.073	9.966	< 0.001
IU1	0.812	0.811	0.045	18.165	< 0.001
IU2	0.889	0.887	0.025	35.194	< 0.001
IU3	0.645	0.643	0.087	7.427	< 0.001
IS1	0.731	0.723	0.022	41.697	< 0.001
IS2	0.798	0.783	0.027	33.201	< 0.001
IS3	0.505	0.523	0.094	5.372	0.041
PEU1	0.811	0.812	0.024	33.558	< 0.001
PEU2	0.707	0.703	0.051	13.950	< 0.001
PEU3	0.883	0.881	0.019	47.623	< 0.001
PEU4	0.736	0.737	0.028	25.918	< 0.001
PEU5	0.737	0.732	0.037	19.737	< 0.001
PU1	0.726	0.727	0.038	18.946	< 0.001
PU2	0.807	0.805	0.024	34.232	< 0.001
PU3	0.773	0.771	0.031	24.864	< 0.001
PU5	0.712	0.712	0.040	17.845	< 0.001
SE1	0.774	0.775	0.031	24.742	< 0.001
SE2	0.870	0.872	0.019	45.064	< 0.001
SE3	0.739	0.735	0.051	14.580	< 0.001

**SD, Standard deviation.**

### Structural Model Assessment

The authors followed the approach proposed by Hair et al. (2019) in order to analyze the structural model. Firstly, the value *R*^2^ for each of the latent variables was obtained to ascertain the in-sample predictive power; secondly, out-of-sample predictive power was assessed through PLS-Predict function in SmartPLS, which uses hold-out sample method. Lastly, bootstrap was run to check for the significance of the path coefficients in the structural model. A 5,000-sampled bootstrap was used for this study, which contained an identical number of observations as the original sample in order to generate the standard errors and *t*-values.

**FIGURE 4:**
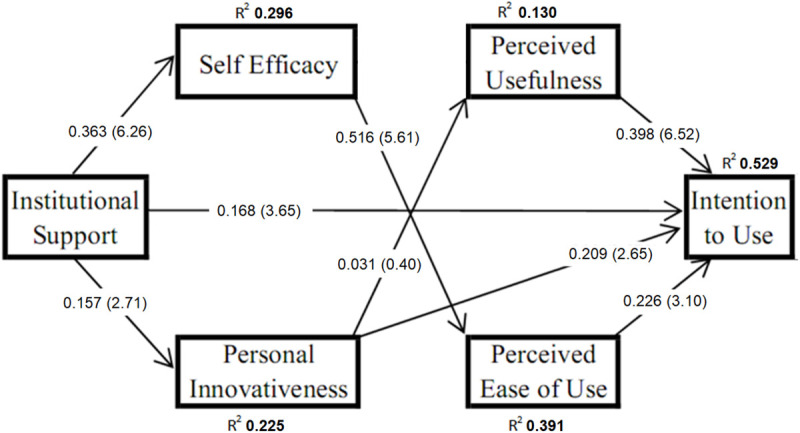
Structural model result.

*R*-square is the in-sample predictive power and the explanatory power of a model. It is the value referred to as the explained percentage of variance in a dependent variable, resulting from the effect(s) of one or more independent variables. Figure 4 shows the structural model results with *R*-square (variance explained) in the endogenous variable. Chin (1998) has recommended *R*-square values of 0.67 for substantial, 0.33 for moderately strong and 0.19 for weak. The analysis shows IS (β = 0.363) explained 29.6% variance in SE; while IS (β = 0.157) explained 22.5% variance in INN. SE has a positive effect on PEU (β = 0.490), explaining 39.1% of its variance. IS, INN, PEU, and PU together explained 52.9% variance in IU.

### Model Quality, Predictive Strength, and Robustness

As recommended by Cohen (1988), *f*^2^-values of 0.02, 0.15, and 0.35 indicate that the interaction term is low, medium, or large on the criterion variable, respectively. A *Q*^2^-value of greater than zero implies that the model has good predictive relevance (Chin, 1998, 2010). The values for *f*^2^ were obtained from the measurement model results, whereas the *Q*^2^-values were obtained through the Blindfolding function under calculate tab of SmartPLS software.

The interaction effect obtained through *f*^2^-values were moderately strong (> 0.15) for interactions between IS and INN, INN and IU, PEU and IU; SE and PEU and this effect was within acceptable level (0.02 or higher) for interactions of IS with IU and SE, as well as for interactions between PU and IU. Whereas the only interaction below the lowest acceptable level of 0.02 was that of INN and PU. The value of *Q*^2^ (i.e., cross-validated redundancy measures) for all the variables was above zero, suggesting predictive relevance is significant. IU and PU had a strong predictive relevance with values of 0.400 and 0.387, respectively, all other constructs also had a moderate predictive relevance with values ranging between 0.232 and 0.300 (Hair et al., 2017; Sarstedt et al., 2017).

In-sample prediction is likely to overstate the model’s predictive ability (than is realistic). It is referred to as an over-fitting problem, indicating the limitation of the model in predicting out of sample observations. PLSpredict function in smartPLS generates holdout based sample predictions of the dependent constructs’ indicators Hair et al. (2019). Following the recommendations by Shmueli et al. (2019), PLSpredict function was run with 10-folds (*k* = 10). Table 4 displays the results which were interpreted by comparing values of RMSE between PLS and linear model (L.M.) because the errors had symmetric distribution (in case of asymmetric distribution, values of mean absolute errors (MAE) should be compared instead of RMSE).

**TABLE 4:** PLS-predict—out of sample predictive power.

**Items**	**PLS-RMSE**	**Q^2^_predict**	**LM-RMSE**	**Difference in RMSE**	**Better predictive validity**
INN1	0.923	0.049	0.936	−0.013	PLS
INN2	0.737	0.001	0.736	0.001	LM
INN3	0.675	0.013	0.682	−0.007	PLS
INN4	0.922	0.004	0.934	−0.012	PLS
IU1	0.564	0.022	0.565	−0.001	PLS
IU2	0.525	0.088	0.542	−0.017	PLS
IU3	0.429	0.181	0.442	−0.013	PLS
PEU1	0.534	0.190	0.576	−0.042	PLS
PEU2	0.473	0.105	0.493	−0.020	PLS
PEU3	0.427	0.247	0.478	−0.051	PLS
PEU4	0.477	0.087	0.493	−0.016	PLS
PEU5	0.458	0.119	0.478	−0.020	PLS
PU1	0.568	0.009	0.563	0.005	LM
PU2	0.554	0.060	0.576	−0.022	PLS
PU3	0.502	0.109	0.534	−0.032	PLS
PU5	0.486	0.092	0.505	−0.019	PLS
SE1	0.553	0.080	0.556	−0.003	PLS
SE2	0.447	0.202	0.451	−0.004	PLS
SE3	0.504	0.094	0.527	−0.023	PLS

**RMSE, root mean square error; LM, linear model.**

The desirable outcome is that all or majority of the items have a lower RMSE value for PLS than L.M. If PLS-RMSE for all dependent indicators is lower than LM-RMSE, the model has high predictive power. If the majority of the indicators have lower RMSE for PLS as compared to the LM-RMSE, the model has medium predictive power. When an equal or a minority of these indicators has lower PLS-RMSE than LM-RMSE, the model has low predictive power. When none of the indicators have lower PLS-RMSE, the model has no predictive power. Table 4 shows that 17 out of 19 items had a lower RMSE for PLS, indicating that the model had a medium predictive power. Table 5 shows model quality results.

**TABLE 5:** Quality and strength of the model.

	***f*-Square values**					***Q*-Square values**		
	**IU**	**INN**	**PEU**	**PU**	**SE**	**SSO**	**SSE**	***Q*^2^ (= 1−SSE/SSO)**
INN	0.181			0.017		567	339.966	0.4
IS	0.084	0.162			0.093	756	566.906	0.25
PEU	0.325					567	416.454	0.266
PU	0.091					756	535.28	0.292
SE			0.271			945	579.641	0.387
IU						567	435.18	0.232

Finally, the structural model assessment was completed by the *t*-value test at 0.05 level of significance calculated using a one-tailed estimation (Hair et al., 2013). Based on the *t*-value rule of thumb for interpretation of a one-tailed test, i.e., 1.65, all the hypotheses were supported with one exception. Table 6 shows the *t*-values and *p*-values, indicating that INN did not prove to be a significant predictor of PU (*t* = 0.40, *p* = 0.339).

**TABLE 6:** Results of structural model and hypotheses testing.

	**β**	**Mean**	**SD**	***T*-values**	***P*-values**	**Hypotheses result**
IS-SE	0.363	0.363	0.058	6.26	< 0.001	H1a = supported
IS-IU	0.168	0.166	0.046	3.65	< 0.001	H1b = supported
SE-PEU	0.516	0.523	0.092	5.61	< 0.001	H2 = supported
INN-PU	0.031	0.036	0.077	0.40	0.339	H3a = not supported
IS-INN	0.157	0.18	0.058	2.71	0.003	H3b = supported
INN-IU	0.209	0.203	0.079	2.65	0.004	
PU-IU	0.398	0.399	0.061	6.52	< 0.001	H4a = supported
PEU-IU	0.226	0.221	0.073	3.10	< 0.001	H4b = supported

*β, path coefficient; SD, standard deviation.*

### Multi-Group Analysis (MGA) Results

Table 7 displays the results of MGA. Respondents were divided into age groups of young (up to 45 years) and old (46 and above); gender (1 = male, 0 = females); less experienced (<5 years) and more experienced (> 5 years); and designation based on seniority (lectures as one group and other senior teaching designations combined as a second group). PLS-MGA was run with a 5,000 bootstrap setting. Benchmarks proposed in the literature state that difference is indicated when the *p*-value differential column displays values below 0.05 or above 0.95 (Ringle et al., 2015). The *p*-values show differences for three paths. The more experienced respondents displayed a stronger SE to PEU path coefficient (β = 0.509, *t*-value = 7.22) as compared to those with lesser experience (β = 0.241, *t*-value = 3.027). For the path IS to INN, respondents also showed differences based on experience. More experienced employees showed a weaker path (β = 0.124, *t*-value = 2.204) as compared to less experienced employees (β = 0.411, *t*-value = 7.918). Differences was also observed for the path IS to SE based on teaching seniority (designations), the most junior designation of lecturers (*n* = 231) showed a weaker path (β = 0.116, *t*-value = 2.192) as compared with other senior teaching designations (*n* = 360) grouped together who showed a higher path coefficient (β = 0.201, *t*-value = 5.372).

**TABLE 7:** Results of the MGA—*P*-values of differences.

	**Males vs. females**	**Young vs. old**	**Less experienced vs. more experienced**	**Lecturers vs. senior faculty**
IS -> IU	0.166	0.816	0.096	0.691
IS -> INN	0.059	0.792	**0.037**	0.931
IS -> SE	0.369	0.076	0.19	**0.032**
SE -> PEU	0.081	0.463	**0.016**	0.066
INN -> PU	0.093	0.063	0.066	0.096
INN -> IU	0.064	0.134	0.336	0.341
PEU -> IU	0.071	0.093	0.191	0.606
PU -> IU	0.096	0.134	0.069	0.393

*Bold values represent significant differences between groups.*

### *Post-hoc* Mediation Analysis

Table 8 displays the significant specific mediation paths. INN mediates relationship between IS and IU as well as IS and SE Moreover, SE proved to be a significant mediator between IS and PEU.As for INN as a mediator, it shows a complementary mediation between IS and IU because IS also has a direct effect on IU Similarly, INN acts a complementary mediator between IS and SE Whereas, SE only has an indirect effect between IS and PEU and PU because IS does not have a direct effect on both PEU and PU Moreover, there is a sequential mediation through the path IS -> SE -> PEU -> IU.

**TABLE 8:** Results of specific indirect effects.

**Specific indirect effects**	**Original sample (O)**	**Sample mean (M)**	**SD**	***T*-statistics (O/SD)**	***P*-values**
IS -> INN -> IU	0.041	0.042	0.018	2.277	0.028
IS -> SE -> PEU	0.158	0.161	0.041	3.853	< 0.001
IS -> SE -> PEU -> IU	0.019	0.021	0.007	2.714	0.019
IS -> SE -> PU -> IU	0.017	0.019	0.006	2.833	0.026

## Discussion

This paper integrates organizational support theory with technology acceptance literature and extends the TAM model with organizational and individual traits from an employee perspective. It investigated IS as a predictor of INN and the level of SE of employees while shaping their perceptions. Moreover, as users of technology, PU and PEU of employees have a strong positive effect on their IU. Thus, it contributes by showing the significant impact of IS in establishing the desired attitude amongst employees toward technology adoption through appropriate measures of institutional support (Zainab et al., 2017). IS may include measures such as awareness sessions, training needs assessment, and incentives for early adopters (Kim and Rha, 2018).

The results affirm that IS enhances the level of SE amongst employees. The level of SE plays a significant role as a mediator between IS and technology adoption. It is consistent with past studies (Igbaria and Iivari, 1995; Al-Haderi, 2013), which proved a significant indirect effect of SE on technology acceptance amongst individuals. Agarwal and Karahanna (2000) and Venkatesh and Davis (2000) both found that an individual’s belief in their ability to use new technology also affects their technology adoption decision. This ability has been conceptualized as SE and has proved with respect to the predictive effect on perceptions; there is a moderate predictive effect of SE on both PEU and PU; which is contradictory to some studies that showed that there was no significant relation between SE and the technology adoption decision of the individuals (Chau and Hu, 2001; Brown et al., 2003; Ozturk et al., 2016). Past research has also argued that under such conditions where it is easy to use a technology or when it is of advantage to the user, the confidence of experienced individuals in their capabilities (self-efficacy) did not affect technology acceptance behavior (Brown et al., 2003) in information system adoption.

The results also show a significant role of IS in enhancing employee INN. IS and INN prove to be part of the mechanism that affects the employees’ IU for EMIS. Support in the form of training and development activities and back-end support by I.T teams helps resolve ambiguities for ongoing technical issues through experience of using information systems at workplace. Thus, leading on to better perceptions toward the use of technology and which also ultimately results in positive intention to use among employees. Moreover, trainings and feedback also inculcate a habit of learning and trying out new ideas and software applications. This habit, when developed over a longer period of time, manifests itself in the shape of innovative behavior where the individual is ready to try new ideas and feels excited to be part of a change process.

Contradictory to our hypothesis, INN did not prove to be a significant predictor of perceived usefulness. However, it did significantly predict intention to use EMIS. The findings that there is no influence of INN on PU is consistent with the findings by Agarwal and Karahanna (2000) and with a more recent study by Joo (2015) wherein INN did not prove to be a significant predictor of PU It contradicts some studies that proved INN as a significant predictor of PU (Lu et al., 2005; Dai et al., 2015; Cao et al., 2019). Moreover, another study by Ham (2009) showed that INN had a negative impact on usefulness. Therefore, there is need for research on contexts wherein INN has differing relationship with user perceptions in technology use.

From a practical point of view, there is growing concern about the need for autonomy of teachers over technology integration in teaching (Fraillon et al., 2014). Based on the propositions of organizational support theory, IS enhances the SE and INN of employees and in turn improves their PU, PEU, and IU toward EMIS. Existence of IS helps in ensuring timely assessment of the need for action because efficient systems of support involve regular and timely feedbacks from employees. Moreover, IS does not only have outgoing effects on employee behavior, it also brings back the benefits to management where exchange of feedback to and from the employee helps refine processes (Zhang et al., 2018). These feedbacks may include suggestions for the pre-implementation plans or the post implementation fixes needed for improving acceptability amongst employees. For any technology to be successful, top management must adhere to the basics of task technology fit concept (Lee et al., 2007; Kim et al., 2010; Liu et al., 2011); ensuring that the technology is well in line with the nature of tasks involved in the day to day routines of the business and is also in line with the employee capability. The employee is at the center of the task technology fit. Employees that exhibit higher levels of SE (Kim et al., 2010) and INN, tend to be vital from the view point of task-technology fit as well. Unlike technologies for personal use, EMIS are used primarily for work-related tasks.

The interaction and communication created by IS helps management identify employees with high INN. These employees, who are potentially early adopters, can be selected as the first batch for the roll out of new technology to ensure successful implementation and developing positive perceptions amongst other employees toward an intended technological change. Thus, IS can help establish necessary steps to bring about the desired attitude amongst employees by using appropriate measures such as awareness sessions, training needs assessment and incentives for early adopters. IS brings about a culture of inclusiveness for employees. It helps the management in identifying SE and INN levels of employees. Further practical implications can be observed from the viewpoint of implementation of modern technology-enhanced education MIS such as Massive Open Online Courses (MOOCs). MOOCs are offered by only a few major platforms where teachers place their customized courses online with self-study materials and tutorial videos.

## Conclusion

The role of employees in successful technology implementation is of primary importance because the user’s acceptance is a critical aspect in an organization (Ahmed et al., 2019; Scherer et al., 2019). IS extends a positive experience accumulation amongst employees leading to positive attitudes. This study adds the effect of organization-level construct of IS on employees’ individual level traits of SE and INN as enablers of acceptance and use of EMIS to the literature. The study generally confirmed the key propositions of TAM. More importantly, the findings show that IS predicts INN and SE; which in turn play a mediating role in creating strengthening perceptions of ease and usefulness to enable positive attitudes and an intention to use EMIS.

This study also has its limitations. First, it is based on a single source data although the researchers used time-lagged data to cater for bias. It is still considered a limiting aspect by most researchers despite being the most common method. Second, although the majority of studies are based on cross-sectional data in TAM studies, causality has been a major question and limiting aspect which is also the case in this study. Three, it assesses the university employees, so it is typically limited to the education sector organizations, which was in fact a contribution of the study as well. Four, the study uses self-reported measures; although this is consistent with past studies, it still may be considered as a limitation. Future studies may include multi-source data instead of single source data to reduce potential for common method variance, for example, supervisor-reported constructs such as innovative work behavior could be included. The difference between junior and senior teaching staff may have been because of other confounding variables beyond the scope of this study. Future studies may involve studying more factors in analyzing difference in their behavior toward EMIS use, such as extrinsic rewards and intrinsic motivation to use EMIS. Another important disclosure for this study is that since the data collection relied on liaison personnel in the universities to ensure that actual users of EMIS were included in the sample and to avoid non-users inclusion in sample; personal relationships of the liaison personnel with the potential respondents could have played a role in selection of respondents, but this could not be controlled under the circumstances as there is no mechanism to identify whether such a bias existed in selection. Moreover, although the non-response bias proved to be non-significant between respondents, care must be taken in drawing general conclusions at the institutional level because some universities did not take part in the survey at all during the first step in the selection process of participating institutions. Thus, it warrants carrying out more similar studies to replicate the results and improve generalizability.

This study also holds the potential for replication in other European countries as there is a 15% growth estimated for e-learning market from 2019 to 2023 indicated in a report by Technovia. The report also states that in 2018, United Kingdom lead the market with 28% share in e-learning, followed by Germany and France (Businesswire, 2019). For example, the number of students registered for distant and online learning programs in Germany exceeded 158,000 in 2016 while an estimated 17,000 learners participated in professional certification programs (Dieckmann and Zinn, 2017). The EMIS and similar systems implementation is an area of interest in this regard.

For future research, it is suggested that (i) this study may be replicated in other developing countries in the Asia–Pacific region where educational information systems are relatively new but are a booming concept; this study has particular implications for other countries like Pakistan, India, Bangladesh, and Sri Lanka where online education and distance learning is on the rise; (ii) for studying additional organizational factors such as leadership styles, empowerment, knowledge diversity, and creative process management amongst university employees and its impact on technology adoption behavior in educational institutions in higher education settings; (iii) it may be replicated for other modern technology-enhanced education systems such as Massive Open Online Courses (MOOCs) and Technological Pedagogical and Content Knowledge (TPACK) which are considered high-end educational technologies.

## Data Availability Statement

The datasets presented in this article are not readily available because the data is part of an ongoing prospective cohort study and institutional permissions restrict sharing the data for 1 year after completion of the project. Requests to access the datasets should be directed to FA, fawadahmed1@live.com.

## Ethics Statement

The studies involving human participants were reviewed and approved by Ethics Committee of School of Management, Wuhan University of Technology. The patients/participants provided their written informed consent to participate in this study.

## Author Contributions

FZ, FA, and MKI: conceptualization. FA, VJH, and YJQ: data collection and curation, and investigation. MKI, MFM, and NAF: formal analysis. FZ: funding acquisition and supervision. MKI, MFM, FA, and VJH: methodology. FA and VJH: project administration. MFM and YJQ: resources. FA: software. FA, VJH, YJQ, MFM, and MKI: writing – original draft. FA and NAF: revising the manuscript.

## Conflict of Interest

The authors declare that the research was conducted in the absence of any commercial or financial relationships that could be construed as a potential conflict of interest.

## Appendix

**TABLE A1:** Questionnaire.

Intention to use	I am willing to use EMIS or software system in the future
	I recommend others to use EMIS or similar educational technology systems
	Using EMIS or similar software is fun and a good idea
Perceived ease of use	Learning to operate EMIS and similar technology is easy for me
	It is easy for me to remember how to perform tasks using technology and computers
	Usage of EMIS and similar technology is clear and understandable
	Using Internet and EMIS software is easy for me.
	Overall, I believe EMIS and similar Technology at my workplace is easy to use
Perceived usefulness	Using EMIS and similar Technology enables me to accomplish targets and goals more quickly
	Using EMIS and similar software and Technology increases my productivity
	EMIS and similar Technology improves performance output
	Using EMIS and similar technology enhances my effectiveness on the job
	I can collaborate easily with customers through EMIS and similar Technology in my office
Innovativeness	I like to try new information technologies
	If I find out about new information technology, I seek ways to experience it
	I am usually one of the first among my colleagues/peers to explore new information technology
	In general, I am reluctant to try new information technologies (Reverse coded)
Self-efficacy	I know how to use Computers, software and related Technologies
	I am confident about using any technology at work
	I feel I am in control when I use I.T/Software and related Technologies for my job tasks
Institutional support	A specific person or group is available for assistance with the computers and related Technologies at my office
	I receive sufficient support from my organization while I use the computers and related Technologies
	Management gave clear instructions on how to use software applications and Technology at work
